# Editorial: Electrobiotechnology Towards Sustainable Bioeconomy: Fundamental, Optimization and Applications

**DOI:** 10.3389/fbioe.2022.901072

**Published:** 2022-04-26

**Authors:** Bin Lai, Sarah Glaven, Hao Song

**Affiliations:** ^1^ Department of Solar Materials, Helmholtz Centre for Environmental Research-UFZ, Leipzig, Germany; ^2^ Naval Research Laboratory, Center for Bio/Molecular Science and Engineering, Washington, DC, United States; ^3^ Key Laboratory of Systems Bioengineering, Frontier Science Center for Synthetic Biology, Ministry of Education, Tianjin University, Tianjin, China; ^4^ Collaborative Innovation Center of Chemical Science and Engineering, School of Chemical Engineering and Technology, Tianjin University, Tianjin, China

**Keywords:** electrobiotechnology, synthetic biology, extracellular electron transfer (EET), redox mediator, thermodynamic modelling, electromicrobiology, high throughput screen

Electrobiotechnology is playing an increasing and essential role in sustaining our future society (Harnisch et al., 2015). It provides a unique approach to overcome the fundamentally biological limitation in biosynthetic processes, namely the redox imbalance caused by the degree of reduction of substrate(s) and products, which significantly restricts microbial growth, product yield and titer in fermentation reactors. Electrobiotechnology is an interdisciplinary approach that integrates microbial energy and redox metabolism using an external electrode, and can (at least partially) uncouple the redox metabolism from the carbon core metabolism and consequently elevate the product yield (Kracke and Krömer, 2014), which can even go beyond the stoichiometric upper limit (Lai et al., 2016). Despite this promising feature, electrobiotechnology still needs significant improvement to attract industrial interest. This is not only limited by the low system efficiency due to a poor quantitative understanding of the process, but is also restricted by the microbial host and product profiles that are currently feasible in electrobiotechnology studies.

Achieving a highly electrogenic active biofilm has been a critical focus in the past decades, and efforts have been made to improve the mass and electron transfer within the biofilm and on the interface between biofilm and electrode or between biofilm and bulk phase. In this research topic, Hu et al. comparatively summarized the current understandings of the electrogenic biofilms of *Shewanella* and *Geobacter*, respectively, from aspects of the lifecycle, structure and heterogeneity of the biofilm and also the parameters that affect the development of active biofilms. This knowledge provides the fundamental basis to understand and then rationally engineer electrode biofilms for improved electrogenic activities.

Planktonic bioelectrochemical systems, where cells interact with an electrode but do not form a biofilm, has attracted more interest in the past years. Soluble redox mediators, either self-secreted or artificially added (Schmitz et al., 2015; Lai et al., 2016; Vassilev et al., 2018), were needed in these cases to facilitate extracellular electron transfer (EET). Using the model strain *Shewanella oneidensis*, Xu et al. screened 14 different mediators and subsequently rationally tuned the redox potential of anthraquinone-2-sulfonate by modifying its function group. As a result, they found the mediator-based EET rate follows the Marcus theory for electron transfer: a lower redox potential led to higher current and coulombic efficiency for a cathodic process. Moreover, the outer membrane proteins OmcA and MtrC were also found to be crucial even for this mediator-based EET pathway.

Not targeting the EET components, Zhang et al. reported another approach to improve the EET rate by tuning the redox metabolism of *Shewanella oneidensis*. Acetate utilization pathway was introduced into the strain, allowing anaerobic oxidation of acetate. Thus, lactate can be metabolized into more oxidized compounds (e.g., CO_2_) than acetate in bioelectrochemical system (BES), leading to more free electrons that can be harvested by the anode. Both the current density and coulombic efficiency were significantly improved for the BESs with the recombinant strain compared to that of the wild type. This study presents a good example of how the cellular redox metabolism can affect the EET rate and how to apply synthetic biology tools to tune cells for target purpose.

A high throughput screening platform is urgently needed for the electrobiotechnology research, to gain a systematic understanding and also optimization of the process. A 96-channel potentiostat was recently reported (Frank et al., 2020), eliminating the biggest capital cost for running parallel BES reactors. Following this development, Kuchenbuch et al., in this research topic, reported a customized 96-well electrochemical microwell plate that allows a relatively high throughput examination of several interesting parameters in parallel. Proof-of-concept studies were done on *Shewanella* and *Geobacter* with different carbon sources in this paper, but such a platform can readily be transferred to 1) screen novel electrogenic microorganisms, 2) optimize working conditions for process engineering, 3) evaluate genetically engineered electroactive bacteria, and so on. Genotyping the culture in the microscale BES reactor was also demonstrated in this paper, while phenotyping in principle would also be feasible by using microscopy-based or high-resolution mass spectrometry-based analytics.

The thermodynamics of electrode-driven biosynthesis is poorly understood (Korth et al., 2016; Gildemyn et al., 2017) but fundamentally constrains the upper boundary of the process. The last paper from this research topic from Wise et al. reported a molecular-scale model to simulate the thermodynamic constraints on electrode-driven microbial protein synthesis from C1 source, which is one of the most promising applications of electrobiotechnology in the near future (Molitor et al., 2019). The results suggested the electrochemically-fueled (*via* H_2_) Wood-Ljungdahl pathway requires the lowest energy inputs for amino acids production for autotrophic microorganisms (only ∼64 MJ/kg) and the energy-to-protein efficiency could reach up to 8.9%, much better compared to the conventional protein productions from soybeans and maize.

In summary, this research topic provides representative examples of how organic chemistry, synthetic biology and thermodynamic modelling can be integrated with electrobiotechnology research, as well as a widely-applicable platform that can facilitate the research progress. Electrobiotechnology is a highly interdisciplinary research field. A holistic and quantitative understanding of the process is critical for its rational improvement.
